# Comprehensive Protein Interactome Analysis of a Key RNA Helicase: Detection of Novel Stress Granule Proteins

**DOI:** 10.3390/biom5031441

**Published:** 2015-07-15

**Authors:** Rebecca Bish, Nerea Cuevas-Polo, Zhe Cheng, Dolores Hambardzumyan, Mathias Munschauer, Markus Landthaler, Christine Vogel

**Affiliations:** 1 Center for Genomics and Systems Biology, Department of Biology, New York University, 12 Waverly Place, New York, NY 10003, USA; E-Mails: rebeccabish@gmail.com (R.B.); nerea.cuevaspolo@gmail.com (N.C.-P.); zhe.cheng@nyu.edu (Z.C.); 2 The Cleveland Clinic, Department of Neurosciences, Lerner Research Institute, 9500 Euclid Avenue, Cleveland, OH 44195, USA; E-Mail: hambard@ccf.org; 3 RNA Biology and Post-Transcriptional Regulation, Max-Delbrück-Center for Molecular Medicine, Berlin-Buch, Robert-Rössle-Str. 10, Berlin 13092, Germany; E-Mails: mathias@broadinstitute.org (M.M.); markus.landthaler@mdc-berlin.de (M.L.)

**Keywords:** DDX6, post-transcriptional regulation, protein interactions, SUMOylation, NUFIP2, FAM195A, FAM195B, stress granules, P bodies, mRNA degradation

## Abstract

DDX6 (p54/RCK) is a human RNA helicase with central roles in mRNA decay and translation repression. To help our understanding of how DDX6 performs these multiple functions, we conducted the first unbiased, large-scale study to map the DDX6-centric protein-protein interactome using immunoprecipitation and mass spectrometry. Using DDX6 as bait, we identify a high-confidence and high-quality set of protein interaction partners which are enriched for functions in RNA metabolism and ribosomal proteins. The screen is highly specific, maximizing the number of true positives, as demonstrated by the validation of 81% (47/58) of the RNA-independent interactors through known functions and interactions. Importantly, we minimize the number of indirect interaction partners through use of a nuclease-based digestion to eliminate RNA. We describe eleven new interactors, including proteins involved in splicing which is an as-yet unknown role for DDX6. We validated and characterized in more detail the interaction of DDX6 with Nuclear fragile X mental retardation-interacting protein 2 (NUFIP2) and with two previously uncharacterized proteins, FAM195A and FAM195B (here referred to as granulin-1 and granulin-2, or GRAN1 and GRAN2). We show that NUFIP2, GRAN1, and GRAN2 are not P-body components, but re-localize to stress granules upon exposure to stress, suggesting a function in translation repression in the cellular stress response. Using a complementary analysis that resolved DDX6’s multiple complex memberships, we further validated these interaction partners and the presence of splicing factors. As DDX6 also interacts with the E3 SUMO ligase TIF1β, we tested for and observed a significant enrichment of sumoylation amongst DDX6’s interaction partners. Our results represent the most comprehensive screen for direct interaction partners of a key regulator of RNA life cycle and localization, highlighting new stress granule components and possible DDX6 functions—many of which are likely conserved across eukaryotes.

## 1. Introduction

The concentrations of cellular proteins are finely tuned by a wide variety of regulatory mechanisms at the level of transcription, translation, and degradation. While transcription regulation is an essential process, some studies indicate that post-transcriptional regulation also plays a large role [1,2,3,4], encompassing for example RNA processing, storage, degradation, and translation. Over a thousand human proteins appear to have RNA binding functions and therefore putative roles in post-transcriptional regulation [5]. However, in-depth knowledge of the molecular functions, targets, and binding sites is still limited to only several dozen RNA-binding proteins (RBPs) [5,6,7]—and an accurate understanding of the regulation of protein expression requires further exploration of the network of post-transcriptional regulators with respect to their localization, interaction partners, and functions.

The mammalian DEAD-box RNA helicase DDX6 (also known as p54/Rck) impacts protein expression in several ways [8,9,10,11]. Current research suggests that DDX6 may be involved in several key functions of RNA metabolism, determining determine whether an mRNA is destined for translation, storage, or decay [12,13,14,15,16]. Mechanistic effects of the yeast DDX6 ortholog, Dhh1p, on gene expression have been well studied, with translation being repressed at the initiation phase in a nutrient-responsive manner [16]. Dhh1p also associates with ribosomes, and inhibits mRNA translation concomitant with the elongation step when tethered to the mRNA [17]. With respect to mRNA decapping, DDX6 is important for assembly of the decapping complex [18], and may stimulate DCP2 activity [15]. DDX6-dependent inhibition of protein synthesis (*i.e.*, translation inhibition and mRNA decapping) is thought to converge in the miRNA-silencing pathway, where efficient miRISC-dependent repression requires DDX6 [19]. Thus, while DDX6 is required for miRNA silencing, the precise manner by which DDX6 is recruited to miRNA targets remains poorly understood [20]. DDX6 has also been linked to human disease both as a proto-oncogene in several types of cancer [21,22,23,24], and as a facilitator of the infection process by a number of viruses including HIV and the hepatitis C virus [25,26,27,28]. However, the exact mechanisms by which DDX6 contributes to these pathogenic processes are yet to be determined.

Both the sequence and functions of DDX6 are conserved in a variety of organisms including *S. cerevisiae* (DHH1) [29], *S. pombe* (STE13) [30], *C. elegans* (CGH-1) [31], *D. melanogaster* (Me31B) [32], *X. laevis* (XP54) [33], and mammals [34]. DDX6 has a complex localization pattern: it stains diffusely in the nucleus and cytoplasm, and localizes both constitutively and during stress to at least two different mRNA-protein (mRNP) structures in the cytoplasm, *i.e.*, stress granules and P-bodies [35,36,37,38]. The multiple niches to which DDX6 localizes likely reflect the multiple molecular mechanisms by which DDX6 influences post-transcriptional gene regulation [39]. In one of these functions, DDX6 enhances mRNA decay via the decapping pathway, a role which has been associated with cytoplasmic structures known as P-bodies or GW bodies [14,35,40]. DDX6 is also involved in the repression of mRNA translation at a step following initiation [16,17,41,42]. Under cellular stress, this translation repressor function has been linked to the storage of mRNAs in cytoplasmic bodies known as stress granules [37]. P-bodies, which are constitutively present in most cell types, have an overlapping but distinct protein composition as compared with stress granules, which are inducibly assembled when global protein synthesis is inhibited in response to stress [43]. Finally, DDX6 can alter protein levels via regulation of microRNA activity [8,9,20,44,45].

Despite much recent investigation [11,13,20,46,47], the mechanisms by which DDX6 carries out these diverse functions are still not well understood. A small number of interactions between DDX6 and other proteins have been characterized in several organisms, but overall the evidence is fragmented and anecdotal. For example, DDX6 is known to interact with the decapping proteins DCP1 and EDC3 in processing bodies (P bodies), forming a complex which mediates de-adenylation dependent mRNA decay [14,35,40,48]. An interaction between DDX6 and the Argonaute proteins, which work in concert with miRNAs to regulate protein levels, has also been posited to occur within P-bodies [44]. DDX6 has further been shown to interact with the translation repressors ataxin-2/ataxin-2 like protein (ATXN2/ATXN2L) and polyadenylate-binding protein 1 (PABPC1) both under normal conditions (where no stress granules are present), and within stress granules under appropriate conditions [37,49].

While these known DDX6 interactions are suggestive of the mechanisms by which DDX6 exerts its influence on post-transcriptional regulation, only a systematic and comprehensive investigation of the protein interaction partners enables us to fully understand the scope and mechanism of DDX6 function. We undertook such an unbiased study of the DDX6-centric protein interactome to shed light on its role in post-transcriptional regulation of cellular protein levels. As a result, we have expanded our knowledge of the proteins involved in DDX6-mediated processes, and identified, verified, and characterized new proteins which re-localize to stress granules under conditions of cellular stress.

## 2. Results

### 2.1. Identification of DDX6-Interacting Proteins

**Figure 1:**
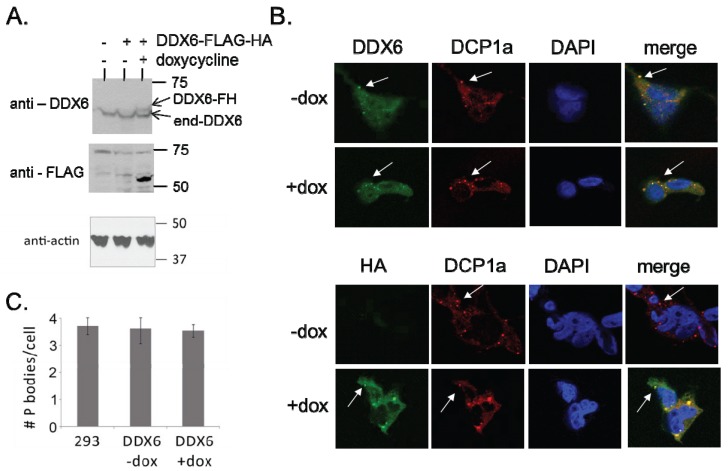
Validation of double-tagged DDX6 construct to ensure highly specific interaction screen. (**A**) Exogenously expressed DDX6 has physiological expression levels. Western blot analysis of whole cell lysates from HEK-293 cells (lane 1) or HEK-293 cells containing the DDX6-FLAG-HA construct under control of a tetracycline-inducible promoter (lanes 2 and 3). DDX6-FH = FLAG-HA-tagged DDX6 construct inducibly expressed from 293-DDX6-FH cells. End-DDX6 = endogenous DDX6; (**B**) Exogenously expressed DDX6 localizes to the expected cellular compartments. Immunofluorescence analysis of HEK-293-DDX6-FLAG-HA cells, in the absence or presence of doxycycline, with antibodies against endogenous DDX6 or the HA epitope tag; (**C**) Quantification of the average number of P bodies per cell in HEK-293 cells as compared to 293-DDX6-FH cells in the absence or presence of doxycycline.

To identify proteins which interact with DDX6, we created an HEK293-based cell line which stably expresses DDX6 fused with an N-terminal FLAG/HA-tag under control of a tetracycline-inducible promoter (293-DDX6-FH). Previous reports indicate that high-level overexpression of DDX6 can induce the formation of additional P bodies, as observed for other P body proteins [16,50]. To avoid this scenario, we selected a clone of the 293-DDX6-FH line which upon induction resulted in expression of DDX6-FLAG-HA at approximately 80% of the level of wild type DDX6 expression (Figure 1A). Repeat experiments demonstrated that this clone reproducibly results in FLAG/HA-DDX6 levels of 70%–90% of endogenous DDX6 levels after 18 h of doxycycline induction. We further confirmed that the DDX6-FLAG-HA protein localizes to P bodies in a manner indistinguishable from that of endogenous DDX6 (Figure 1B), and that the average number of P bodies per cell is unchanged by DDX6-FLAG-HA expression (Figure 1C). These results suggest that the DDX6 transgene is fully functional in terms of localization, and does not alter cellular mRNP composition.

**Figure 2:**
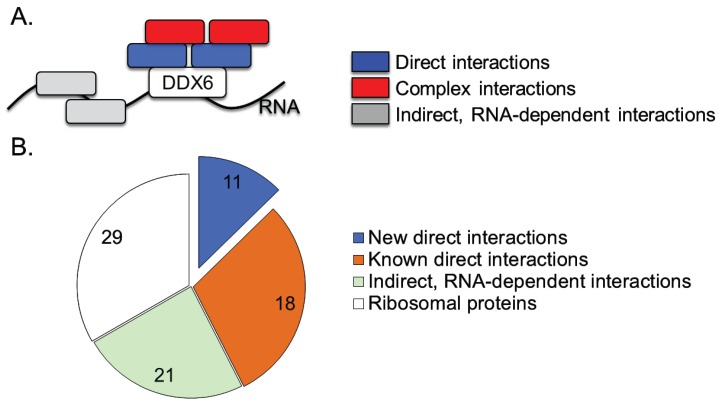
DDX6-interacting proteins belong to three major categories. (**A**) Graphical representation of interaction types identified in this screen. Blue—direct interactions, enriched in pulldown of double-tagged DDX6. Red—complex interactions, identified in native gel experiment. Grey—RNA-dependent interactions; (**B**) DDX6-interacting proteins identified in this screen. The novel, non-ribosomal proteins, likely to be direct interaction partners, are emphasized in blue. Each pie wedge is labeled with the number of proteins identified in that category.

We employed two major approaches to characterize both the direct and indirect interaction partners for DDX6 (Figure 2A). First, we used the double-tagged DDX6 protein as bait in a tandem-pulldown experiment which, due to the two consecutive purification steps is likely to be enriched in true-positive interaction partners. To do so, we harvested whole cell lysate from 293-FLAG-HA cells treated with doxycycline, and performed a tandem immunoprecipitation (IP) against the FLAG and HA epitope tags. RNA-dependent interactions, *i.e.*, proteins bound to the same RNA but not to DDX6, were removed by benzoase treatment, a highly active nuclease which nonspecifically degrades both RNA and DNA. Identical experiments performed in doxycycline-treated HEK293 cells lacking the DDX6-FLAG-HA construct served as a control for non-specific binding. The purified proteins were digested with trypsin and the resulting peptides were identified by mass spectrometry. Second, in a complementary approach, we used single-pulldown of DDX6 and native-gel electrophoresis to separate different protein complexes involving DDX6. Importantly, our protocol involves a benzoase digestion step which removes all RNA. Therefore, in contrast to many other studies, e.g., [51], our results report very few false-positive interaction partners, *i.e.*, proteins that are purely reported due to their binding to the same RNA.

Several criteria were applied to ensure the validity of the list of DDX6-interacting proteins. We applied a 5% false discovery rate cutoff to each individual replicate, and included only proteins which were identified by at least one peptide in a minimum of two biological replicates of one experimental condition (control HEK293 *vs.* experimental 293-DDX6-FH IP) on the list. Furthermore, any protein identified by a single peptide in the control IP was considered a contaminant and excluded from the list, as were all keratins, histones, and immunoglobins. These strict criteria ensure high confidence in the identified proteins.

Applying these criteria, a total of 81 proteins were identified in the tandem-pulldown experiment, including DDX6 itself (Supplementary data). We subdivided the list into several categories (Figure 2B). As could be expected, we identified more RNA-dependent than independent interactions (Supplementary data). The 23 RNA-dependent proteins are enriched in RNA-binding functions (GO: 0003723), and likely identified due to binding of a common RNA molecule, without maintaining a direct protein-protein interaction. In agreement with this interpretation, none of the RNA-dependent DDX6 interactors had previously been identified as directly binding to DDX6.

A second group of proteins identified as interacting with DDX6 is composed of ribosomal proteins from both the large and small ribosomal subunits (Supplementary data and Figure 2B). A physical interaction between DDX6 and the ribosome is not surprising, given the previously documented interaction of the yeast ortholog DHH1 with the ribosome [17], and the documented role of DDX6 in the regulation of translation efficiency [16,17]. In the interest of focusing on previously uncharacterized DDX6 protein partners, interactions with the ribosome will not be further discussed here.

We identified 29 non-ribosomal proteins as interacting with DDX6, independent of RNA presence, and these proteins represent a core which we examined in more detail (Table 1 and Figure 2B). Amongst the proteins, several functional categories were significantly enriched: RNA binding (*p* = 4.7 × 10^−8^), deadenylation-dependent mRNA decay and P-bodies (*p* = 2.2 × 10^−7^), and cytoplasmic stress granules (*p* = 3.2 × 10^−7^). These categories are in accordance with the known functions of DDX6 in mRNA decay, and its localization to cytoplasmic stress granules [52]. There is a high degree of interconnectivity within the DDX6 interactome, as 25 of the 29 DDX6-interacting proteins identified in this study have been shown to bind at least one other protein within this group (Supplementary data).

A literature search reveals that 18 of the proteins have previously been shown to interact with DDX6: the ATXN2/ATXN2L-PABPC1 complex, the DCP1-EDC3-PAT1L decapping complex, LSM14A, transcription intermediary factor 1-beta (TIF1B), and eukaryotic translation initiation factor 4E transporter (EIF4ENIF1), FAM195A and FAM195B [37,40,48,49,52,53,54]. For several other proteins, *i.e.*, LSM14B, EDC3, NUFIP2, other large-scale screens reported possible complex or indirect interactions (Table 1). Other proteins are known to be components of stress granules, P-bodies, or the decapping complex which are all functions that are known to involve DDX6 (Table 1). These known interactions and functions to verify that our interaction screen is highly specific.

In total, eleven (19%) of the identified proteins represent novel interactions with DDX6 in the context of human cells that have the potential to provide insight into DDX6-mediated cellular processes (Table 1). Six of these new interactors, namely C1QBP, DDX17, HNRNPC, HNRNPM, RTCB, and THRAP3, are annotated as splicing factors—a function that DDX6 has not previously been implicated in.

**Table 1:** Interaction partners, independent of binding to RNA.

Gene Name	Protein Name	SG	TL	PB	DC	Status	SF
ATXN2	ataxin 2	X	X	X		BG	X
ATXN2L	ataxin 2-like	X		X		BG	X
C1QBP	complement component 1, q subcomponent binding protein					New	X
DCP1B	DCP1 decapping enzyme homolog B			X	X	BG	
DDX1	DEAD (Asp-Glu-Ala-Asp) box helicase 1	X	X			Inf	X
DDX17	DEAD (Asp-Glu-Ala-Asp) box helicase 17					New	X
EDC3	enhancer of mRNA decapping 3 homolog			X	X	BG	
EDC4	enhancer of mRNA decapping 4			X	X	BG	
EIF4ENIF1	eukaryotic translation initiation factor 4E nuclear import factor 1					BG	
ERH	enhancer of rudimentary homolog (Drosophila)					New	
FMR1	fragile X mental retardation 1	X	X			New	
FXR2	fragile X mental retardation, autosomal homolog 2		X			New	
G3BP2	GTPase activating protein (SH3 domain) binding protein 2	X				BG	
GRAN1/ FAM195A	family with sequence similarity 195, member A	X***				BG	
GRAN2/ FAM195B	family with sequence similarity 195, member B	X***				BG	
HNRNPC	heterogeneous nuclear ribonucleoprotein C (C1/C2)					New	X
HNRNPM	heterogeneous nuclear ribonucleoprotein M					New	X
LARP4	La ribonucleoprotein domain family, member 4					New	
LSM12	LSM12 homolog					BG	
LSM14A	LSM14A, SCD6 homolog A	X	X	X		BG	
LSM14B	LSM14B, SCD6 homolog B		X			BG	X
NUFIP2	nuclear fragile X mental retardation protein interacting protein 2	X***				BG	
PABPC1	poly(A) binding protein, cytoplasmic 1	X	X			Inf	X
PABPC3	poly(A) binding protein, cytoplasmic 3	X				Inf	
PABPC4	poly(A) binding protein, cytoplasmic 4 (inducible form)	X	X			Inf	X
PATL1	protein associated with topoisomerase II homolog 1			X	X	BG	
RTCB	RNA 2',3'-cyclic phosphate and 5'-OH ligase					New	X
THRAP3	thyroid hormone receptor associated protein 3					New	X
TIF1B	tripartite motif containing 28					New	

Proteins identified by mass spectrometry from samples generated by immunoprecipitation of DDX6 from nuclease-treated samples. Status—status of interaction (New—interaction partner newly detected in this screen; Inf—interaction can be inferred from DDX6’s new function in DC, SG, TL, or PB; BG—interaction with DDX6 reported in Biogrid [55]). SG—stress granules. TL—translation. PB—processing bodies. DC—decapping complex. SF—splicing factor. ***—as demonstrated in this paper.

### 2.2. Validation and Characterization of Direct Interactions with NUFIP2, GRAN1 (FAM195A), and GRAN2 (FAM195B)

**Figure 3:**
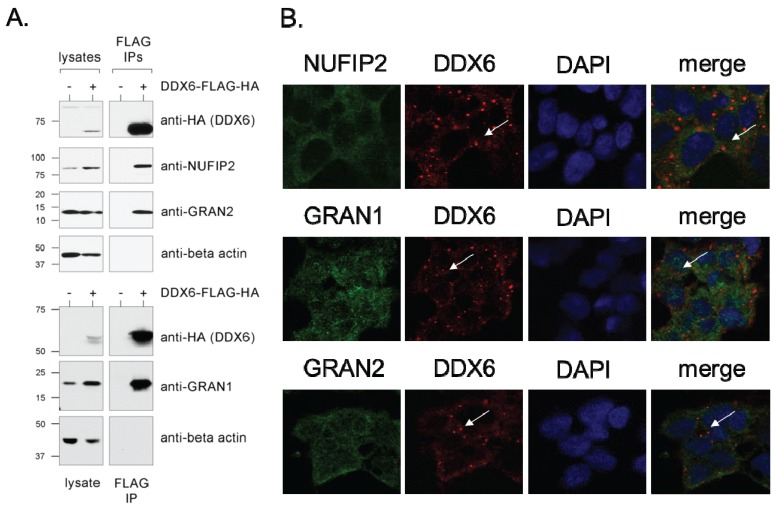
Validation of direct interactions—characterization of new genes. (**A**) DDX6 interacts with NUFIP2, GRAN1, and GRAN2. Whole cell lysate (lanes 1 and 2) or tandem FLAG/HA immunoprecipitates (lanes 3 and 4) were probed by Western blot with antibodies against the HA epitope tag, NUFIP2, GRAN1, GRAN2 or beta-actin (loading control). The experiment was performed in HEK-293 cells (control) or in 293-DDX6-FH cells induced with doxycycline as indicated; (**B**) NUFIP2, GRAN1, and GRAN2 do not localize to P bodies. For the co-localization of DDX6 with GRAN1, GRAN2, and NUFIP2, cells were double-stained with an antibody against DDX6 (red) and the interacting proteins as indicated (green). Nuclei are marked with DAPI. White arrows indicate the location of a DDX6-positive P body.

We chose three of the DDX6-interacting proteins for further validation and characterization as very little or nothing was known about their function: NUFIP2, FAM195A, and FAM195B. FAM195A and FAM195B have recently been listed as DDX6 interaction partners in a large-scale experiment [53], but this result has not yet been validated or characterized. Due to results that will be discussed below, we have renamed the genes as granulin-1 (GRAN1, FAM195A) and granulin-2 (GRAN2, FAM195B). NUFIP2 has also not been well-characterized, but has been shown to associate with Fragile X Mental Retardation Protein (FMR1, also identified as DDX6-interacting protein in this study) and the polysome [56,57]. First, to confirm the interactions with DDX6, we performed an immunoprecipitation using anti-FLAG antibodies in lysate prepared from 293-DDX6-FH cells, and subjected the purified proteins to Western blot with antibodies specific to NUFIP2, GRAN1, and GRAN2 (Figure 3A). For all three antibodies, a band was identified at the correct molecular weight in whole cell lysates. A similar band was observed when blotting proteins purified specifically in the IPs from 293-DDX6-FH cells but not the control HEK-293 cells, validating the interactions observed by mass spectrometry.

We then examined the co-localization of NUFIP2, GRAN1, and GRAN2 with DDX6 by performing immunofluorescence experiments in HEK-293 cells with antibodies specific to each protein. All three proteins showed dual localization to the cytoplasm and nucleus, and they exhibit diffuse staining both in the nucleus and cytoplasm which overlap with the diffuse DDX6 staining (Figure 3B). However, none of the three proteins stain within P bodies, as can be seen by the lack of co-localization in the DDX6-positive cytoplasmic foci (Figure 3B).

**Figure 4:**
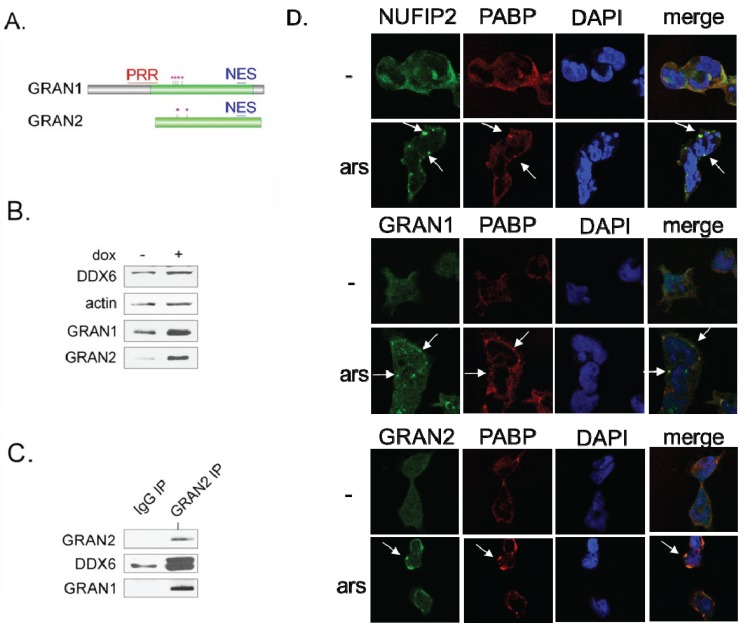
GRAN1, GRAN2, and NUFIP2 localize to stress granules upon arsenite treatment. (**A**) Domain structure and features of GRAN1 (FAM195A) and GRAN2 (FAM195B). Green = FAM195 domain [61]. PRR = proline-rich region. NES = nuclear export signal [62,63]. Purple stars = serine/threonine phosphorylation sites from the PhosphoSite database [58]; (**B**) GRAN1 and GRAN2 protein levels increase upon induction of DDX6 overexpression. Whole cell lysates from 293-DDX6-FH in the absence or presence of doxycycline were separated by SDS-PAGE and blotted with the indicated antibodies; (**C**) GRAN1 and GRAN2 form a physical complex. An immunoprecipitation experiment with an anti-GRAN2 antibody was subject Western blot with antibodies that recognize GRAN2 and DDX6; (**D**) NUFIP2, GRAN1, and GRAN2 localize to stress granules. HEK293 cells were either mock-treated (-) or subject to arsenite (ars) treatment (50 mM, 1 h) prior to immunofluorescence staining with antibodies against NUFIP2, GRAN1, or GRAN2. Stress granules were identified by staining with the stress granule marker PAPB, and DAPI marks the location of the nucleus. White arrows indicate stress granules.

GRAN1 and GRAN2 are small proteins distinguished primarily by the presence of the FAM195 protein domain (Figure 4A). They have not yet been described in literature. Mass spectrometry data indicates that both GRAN1 and GRAN2 possess a similar cluster of phosphorylation sites in the N-terminal side of the FAM195 domain (Figure 4A) [58]. GRAN1 and GRAN2 protein levels appear to be influenced by DDX6 expression, as induction of DDX6 overexpression in 293-DDX6-FH cells by doxycycline treatment induced a significant increase in GRAN1 and GRAN2 levels compared to the loading control of beta-actin, as measured by Western blotting of whole cell lysates (Figure 4B). This increase in GRAN1 and GRAN2 expression could happen through a number of different mechanisms, including translational regulation or stabilization of GRAN1 and GRAN2 protein levels via complex formation with DDX6.

Next, because of their similar localization patterns, we tested whether GRAN1 and GRAN2 interact by immunoprecipitating GRAN2 from HEK293 cells and blotting for GRAN1 and DDX6 (Figure 4C). This experiment indicates that GRAN1 and GRAN2 do interact, and also confirms the DDX6 interaction via a reciprocal co-immunoprecipitation.

We next examined the subcellular localization pattern of GRAN1, GRAN2, and NUFIP2, to gain additional insight into their potential functions. DDX6 undergoes a change in subcellular localization after exposure of cells to various types of stress that induce the formation of stress granules [37,38]. DDX6 can still be found in P bodies under these conditions, but some fraction of DDX6 protein also re-localizes to stress granules. We hypothesized that, since NUFIP2, GRAN1, and GRAN2 are not found in P bodies, they might instead localize to stress granules under the appropriate conditions. This hypothesis is strengthened by the observation that both NUFIP2 and GRAN1 contain Q/N-rich regions (Figure 4A) which have been shown in other proteins to mediate localization to cytoplasmic mRNP particles such as stress granules [59]. Furthermore, NUFIP2 precipitates from cells after treatment with biotinylated 5-aryl-isoxazole-3-carboxyamide, a compound which induces the precipitation of many RNA binding proteins which are constituents of mRNP granules [60].

To test the hypothesis that GRAN1, GRAN2, and NUFIP2 might localize to other mRNP granules, we treated HEK293 cells with arsenite, a reagent known to induce stress granules. We then performed double-immunofluorescence imaging to co-localize these proteins with PABPC1, a well-characterized marker of stress granules (Figure 4D). GRAN1, GRAN2, and NUFIP2 all exhibited a dramatic change in sub-cellular localization upon arsenite treatment, forming cytoplasmic foci that perfectly overlap with stress granules (Figure 4D). These data, combined with their interaction with DDX6, suggests a role for these three proteins in granule formation after stress.

### 2.3. Further Functional Characterization of the DDX6 Interactome

**Figure 5:**
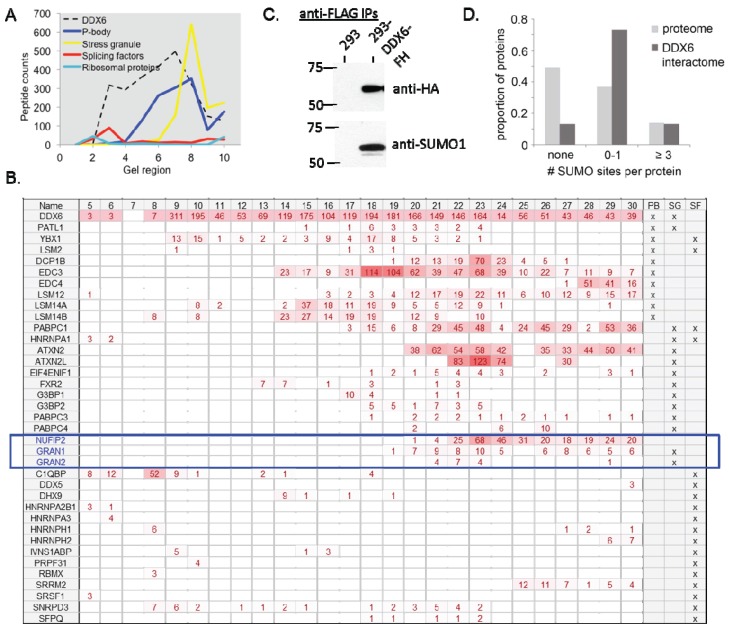
Functional characterization of the DDX6 interactome—complex interactions and SUMOylation. (**A**) Analysis of DDX6-protein complexes by native gel electrophoresis shows that the different functional groupings of DDX6-interacting proteins migrate at different rates, indicating the possibility of distinct DDX6 complexes with different cellular functions. Gel regions represent the combined results from three adjacent gel slices. Proteins with known exclusive membership to a functional class localization (SF—Splicing Factor, PB—Processing bodies, SG—Stress Granules) were grouped and unique peptide counts totaled for the group; (**B**) Distribution of proteins across slices from native gel electrophoresis. We report the number of peptides found for each protein in each slice and their localization. Functional categories as in (A). Highlighted in blue are NUFIP2, GRAN1, and GRAN2 which are further validated and characterized here. The complete dataset is shown in the Supplementary data; (**C**) DDX6 was immunoprecipitated from HEK293 or 293-DDX6-FH cells via a tandem FLAG-HA immunoprecipitation, separated by SDS-PAGE, and blotted with antibodies against SUMO1 and the HA tag to identify sumoylated forms of DDX6; (**D**) DDX6-interacting proteins are significantly enriched in the number of sumoylation sites per protein (t-test, *p* = 4.1 × 10^−5^).

Based on the data discussed above (Table 1) and literature evidence, we hypothesized that DDX6 belongs to several distinct multi-protein complexes, playing a role in different aspects of RNA metabolism (e.g., decapping, splicing, translation regulation, transport), is localized to different cellular compartments (e.g., P bodies, stress granules, diffuse cytoplasmic and nuclear staining). To test this hypothesis, we purified DDX6-protein complexes by immunoprecipitation from 293-DDX6-FH cells, and separated the intact protein complexes by blue native polyacrylamide gel electrophoresis [64,65,66]. The gel lanes from control (HEK293) or 293-DDX6-FH samples were cut into thirty 2 mm slices, and the protein contents were analyzed by mass spectrometry. Identified proteins were classified as either P-body, stress granule, or splicing factors (Figure 5A,B).

We then plotted the number of unique peptides identified in each group according to the gel region which roughly corresponds to the molecular weight of the intact protein complex. Proteins with ambiguous group membership were excluded. In this graph, we note a single peak of splicing factors in the lower molecular weight region (corresponding to approximately 150–300 kD), accompanied by elution of the ribosomal proteins (Figure 5A,B). Notably, elution of splicing proteins is dominated by C1QBP, a protein involved in regulation of RNA splicing by inhibiting the RNA-binding capacity of SRSF1 and its phosphorylation [67]. Is required for the nuclear translocation of splicing factor U2AF1L4 [67]. An interaction with DDX6 has not yet been described.

In the middle molecular weight regions, only the P body proteins are present. In addition, at high molecular weights, both P body and stress granule proteins form an overlapping peak (Figure 5A,B). The co-elution of P-body and stress granule components illustrates the overlapping functionalities and protein membership of these protein complexes. The elution of NUFIP2, GRAN1, and GRAN2 with other known members of stress granules confirms our findings from above on these proteins being new stress granule components (Figure 5B). The separation of protein complexes by molecular weight according to function suggests that DDX6 likely functions in a number of different protein complexes with different functions.

One of the novel DDX6-interacting proteins, TIF1β (Table 1), has recently been identified as an E3 ligase for the small ubiquitin-like protein SUMO [68,69]. Indeed, in one high-throughput study that used mass spectrometry to identify SUMO-conjugated proteins, DDX6 is identified as a sumoylation substrate [70]. We therefore set out to confirm whether DDX6 is sumoylated. We purified DDX6 from 293-DDX6-FH cells by immunoprecipitation, and blotted with an antibody against SUMO1. We observed a band at approximately 65 kD (Figure 5C), leading to the conclusion that DDX6 is sumoylated.

We then hypothesized that other members of the DDX6 interactome might be similarly sumoylated. A recent study lead by Mann’s group and many other studies that use proteomic approaches to identify sumoylated proteins independently concluded that the identified SUMO-conjugated proteins are highly enriched for proteins involved in RNA metabolism and RNA binding [70,71,72,73,74,75,76,77,78,79]. To test whether DDX6-interacting proteins are enriched for sumoylation, we compiled a database of human proteins which have been identified as sumoylated from 14 high-throughput mass spectrometry studies [70,71,72,73,74,77,80,81,82,83,84,85,86,87] (Supplementary data). We included a diverse array of studies, including purifications of SUMO1, SUMO2, and SUMO3, as well as varied purification techniques and experiments performed under stress conditions such as heat shock or oxidative stress, to obtain as wide a representation of the sumoylated universe as possible. In total, 1892 proteins from these studies could be mapped to unique gene identifiers. Of these proteins, 853 were identified by at least two independent studies, and therefore should be considered a core set with especially strong evidence of sumoylation. Of the 29 proteins that we identified as interacting with DDX6 (Table 1), 13 were found to be members of this core set of sumoylated proteins, representing a highly significant enrichment in comparison to the human proteome (hypergeometric test, *p* = 3.4 × 10^−11^) (Figure 5C). This enrichment remains significant even when considering the set of all proteins detected in sumoylation studies instead of the entire proteome (19 of 29, *p* = 5.6 × 10^−9^). In fact, one of the interactors, heterogeneous nuclear ribonucleoprotein M (HNRNPM), is one of the two most frequently-identified sumoylated proteins known, occurring as a hit in ten of the 14 studies examined (Supplementary data). Conversely, analysis of the human proteome with SUMOsp software to identify high confidence sumoylation sites (both Ψ-K-x-E motif and non-consensus sites) found that the DDX6-interacting proteins identified in this paper contain significantly more sites than would be expected given their frequency in the proteome as a whole (Figure 5D, *p* = 4.1 × 10^−5^).

## 3. Discussion

An extensive body of literature documents the participation of DDX6 in a number of cellular processes, all of which contribute to post-transcriptional regulation and many of which are conserved throughout evolution [16,88,89]. However, the mechanisms by which a protein can act as a regulator are mostly based on interactions with other molecules. To help our understanding of the complex regulatory roles of DDX6, we have undertaken the first comprehensive DDX6-centric interaction screen, and characterized the protein interaction partners in a large-scale, comprehensive manner. To accurately and sensitively identify these proteins, we chose a tandem affinity purification strategy of DDX6 as the bait, coupled to high-resolution mass spectrometry. By combining different approaches, we distinguished functionally relevant interactions with DDX6 from interactions which depend on the mutual binding of RNA, and protein complex membership.

DDX6 is often thought of as a marker of P-bodies [90], and the most well-characterized function of both DDX6 and its yeast ortholog DHH1 is in the mRNA decapping/decay pathway [14,47,88,89,91]. Consistent with this view, we identified a number of P-body/decapping proteins as DDX6 interactors (Table 1). Many of these interactions had previously been observed either in human or yeast in individual studies emphasizing the conservation of DDX6’s role across organisms [20,37,46,49,62]. We also identified a number of known and novel interacting proteins which localize to stress granules. Because these experiments were performed under non-stressed conditions in which stress granules are not visible by microscopy, we conclude that DDX6 maintains stable interactions with stress granule proteins even in the absence of visible mRNP structures. Overall, the sheer number of P-body and stress granule proteins with which DDX6 interacts, in addition to the known mRNP remodeling function of DDX6, suggests that DDX6 may be a key factor in modulating the contents of P bodies and stress granules. Indeed, a recent publication [92] demonstrates a key role of DDX6 in P-body assembly.

Notably, we did not identify any peptides from Argonaute proteins, even below the threshold for inclusion in our lists, despite evidence in the literature that such an Ago-DDX6 interaction does occur [44]. This finding is likely due to either the interaction being transient and not stable enough to persist during the tandem immunoprecipitation protocol, or the interaction taking place under conditions not tested here, or the interaction occurring at a level undetectable by mass spectrometry in the quantities used for our experiment. As we placed our screen’s emphasis on minimal false-positives, *i.e.*, low number of falsely reported interaction partners, rather than minimal a few false-negatives, *i.e.*, identification of all known partners, absence of the Argonaute proteins does not diminish the quality of our results. A list of proteins reported to interact with DDX6, but not observed in this study, is provided in the Supplementary Material.

Recent studies identified a physical protein interaction network associated with miRNA biogenesis and regulation, using a proteomic approach [46,53]. The authors listed DDX6-interacting proteins, including NUFIP2, FAM195A and FAM195B, but without further validation. As very little is known about the three proteins, we confirm the interaction and we characterized the function of these proteins with respect to RNA-protein complex interactions. GRAN1 and GRAN2 bind DDX6 and are localized to the cytoplasm under normal conditions. Upon stress, GRAN1 and GRAN2 re-localize to stress granules, suggesting a potential role in translation repression in response to stress and presenting new stress granule components. This dynamic localization to stress granules may be facilitated by the Q/N-rich region in GRAN1. Due to their aggregation-prone nature, Q/N rich regions can be sufficient for localization of a variety of human proteins to P bodies [59,60]. As stress granule proteins also tend to have Q/N rich regions (e.g., ATXN2), the sequence may serve a re-localization function as well. Because GRAN1 and GRAN2 also interact with each other, GRAN2 could be recruited to stress granules via its interaction with GRAN1. In contrast to other interaction partners also reported in yeast, GRAN1 and GRAN2 have orthologs in other vertebrates, but not invertebrates or unicellular eukaryotes—suggesting that their role with DDX6 evolved only recently.

The stress granule protein NUFIP2 is another novel DDX6 interaction partner which we characterized further. NUFIP2 was originally identified through its interaction with the Fragile X mental retardation protein FMR1, but nothing else is known about the protein. Interestingly, FMR1 and its homolog FXR2 were also identified as interacting with DDX6 in this screen, suggesting a role of FMR1/FXR2 in stress that has not been described before. However, given the low relative quantification of each of these proteins in our experiments, the interaction between DDX6 and FMR1/FXR2 may be transient or indirect and mediated by the much more abundant NUFIP2. Both DDX6 and the FMR proteins are noted for their roles in translation repression, and in Drosophila, these proteins have been found to co-localize in neuronal granules, suggesting that they can work in the same pathway [93]. The common interaction with NUFIP2 suggests that further study of this protein may shed light on the nature of translation repression in stress granules. As a side note, NUFIP2 also contains a Q/N rich region (as well as a proline-rich region), which might explain its localization to stress granules.

Studies of protein-protein interactions can often identify potential new functions for a protein through analysis of the known functions of its interaction partners. Table 1 and Figure 5A,B show several proteins which function in RNA splicing. DDX6 has not previously been associated with splicing, and these data suggest a novel role for DDX6 that may explain its presence in the nucleus. Supporting this hypothesis, several mass spectrometry studies in yeast and human have identified DDX6 as a potential component of the spliceosome [94,95,96]. This observation could be explained by the fact that many nuclear mRNP that co-localize in P-bodies and stress granules during stress include factors involved in transcription, 3' end processing, splicing and export, which might affect also nuclear events [97].

Finally, we also detected an interaction between DDX6 and the E3 SUMO ligase TIF1β. All evidence established to date says that TIF1β is an exclusively nuclear protein [98]. However, while DDX6 is primarily cytoplasmic, there is evidence that it shuttles between the nucleus and cytoplasm, creating an opportunity for sumoylation by TIF1β [99]. We confirmed that DDX6 is indeed sumoylated (Figure 5C) [70], and also demonstrate significant SUMOylation for the DDX6 interactome (Figure 5D). One intriguing hypothesis for the mechanism of SUMOylation amongst these RNA-binding proteins is based on the fact that SUMO is one of the most soluble of all known proteins [100], and it may be involved in preventing aggregation within densely packed cellular structures such as these granules [101]. As many or most of the DDX6-interacting proteins localize to P bodies and/or stress granules, and this localization is known to depend on a variety of aggregation-prone domains, we hypothesize that maintenance of a certain level of sumoylation of these proteins prevents uncontrolled aggregation during the assembly of cytoplasmic mRNP granules. Uncontrolled mRNP assembly via these low complexity domains has been posited to contribute to a variety of neurodegenerative disorders [102].

## 4. Experimental Section

### 4.1. Generation of Cell Lines

pENTR constructs were generated by PCR amplification of the respective coding sequences (CDS) from Flp-In 293 T-REx derived cDNA, followed by restriction digest and ligation into pENTR4 backbone (Invitrogen, Carlsbad, CA, USA). pENTR vectors carrying CDS were recombined into pFRT/TO/FLAG/HA-DEST destination vector using GATEWAY LR recombinase according to manufacturer’s protocol (Life Technologies, Carlsbad, CA, USA). Cell lines stably expressing FLAG/HA-tagged DDX6 protein were generated by co-transfection of pFRT/TO/FLAG/HA constructs with pOG44 into Flp-In 293 T-REx cells (Life Technologies). Cells were selected by exchanging zeocin for 100 µg/mL hygromycin, and monoclonal colonies were isolated.

### 4.2. Cell Culture

HEK-293 cells (ATCC) and derivatives were cultured in a 37 °C incubator with 5% CO_2_, in DMEM (Sigma, St Louis, MO, USA) supplemented with 10% FBS (Atlanta Biologicals, Norcross, GA, USA) and 1% penicillin-streptomycin-Fungizone (Life Technologies). For arsenite treatment, cells were incubated with 50 mM sodium (meta)arsenite (NaAsO_2_, Sigma) for 1 h. For DDX6-FLAG-HA induction, cells were treated with 1 µg/mL doxycycline (Sigma) for 18 h.

### 4.3. Immunoprecipitation

For each sample, one 15 cm plate of cells at 80% confluency was rinsed with cold PBS, scraped into 2 mL NP-40 buffer (50 mM HEPES pH 7.5, 150 mM KCl, 2 mM EDTA, 1% NP40, one Complete mini protease inhibitor tablet and one PhosStop tablet per 10 mL (Roche, Basel, Switzerland)) and incubated on ice for 20 min. Protein lysates were then spun at 14,000 RPM for 10 min at 4 °C, and supernatant was used as the protein sample. The lysates were first incubated for 1 h at 4 °C with anti-FLAG agarose beads (Sigma) for the tandem immunoprecipitation, or with Protein G Dynabeads (Life Technologies) bound to an antibody recognizing GRAN2 (Sigma, HPA045542). The beads were washed 3 times in wash buffer (50 mM HEPES pH 7.5, 150 mM KCl, 2 mM EDTA, 0.5% NP40). At this point, some samples were incubated for 10 min at room temperature with 250 units benzonase (Sigma). For the tandem immunoprecipitation, protein complexes were eluted from the beads by 2 incubations with 3XFLAG peptide (Sigma, 100 µg/mL) for 5 min each. The resulting eluate was incubated with Protein G Dynabeads bound to an anti-HA antibody (Sigma, H3663) for 45 min at 4 °C. The bead-bound proteins were then washed 3 times with wash buffer, and either boiled in Laemmli buffer for Western blot (Biorad, Hercules, CA, USA), or washed 3 more times in 25 mM ammonium bicarbonate (ABC) for preparation for mass spectrometry analysis.

### 4.4. Western Blot

Lysates were prepared as for immunoprecipitation, using RIPA buffer (50 mM Tris pH 7.5, 1% NP40, 0.1% SDS, 0.5% sodium deoxycholate, 150 mM NaCl, one Complete mini protease inhibitor tablet and 1 PhosStop tablet per 10 mL) in place of NP-40 buffer. Equal protein amounts were run on SDS-PAGE gels, transferred via wet electroblotting, and blocked in 5% milk in TBS-T (50 mM Tris pH 7.5, 150 mM NaCl, 0.1% Tween-20). The following primary antibodies were incubated with the blots overnight at a dilution of 1:1000 unless otherwise noted: anti-DDX6 (Abcam, Cambridge, United Kingdom, 4967), anti-FLAG (Sigma, F3165), anti-beta-actin (Cell Signaling, Beverly, MA, USA, 4967), anti-HA (Sigma, H6908), anti-NUFIP2 (Sigma, HPA017344, 1:250), anti-GRAN2 (Sigma, HPA045542, 1:250), anti-GRAN1 (ProteinTech, Chicago, IL, USA, 20808-1-AP), anti-SUMO1 (Abcam, ab32058). HRP-conjugated secondary antibodies were used at a dilution of 1:5000 for 1 h (GE Healthcare, Little Chalfont, UK).

### 4.5. Blue Native Polyacrylamide Gel Electrophoresis

A single anti-FLAG immunoprecipitation step was performed as described above, including elution with the 3X FLAG peptide. Eluates were separated using the NativePAGE system (Life Technologies) according to the manufacturer’s instructions, on a 1 mm 3%−12% Bis-Tris gel. Gel bands from the entire lane were cut with a width of 2 mM from a GelCode Blue-stained gel (Pierce Biotechnology, Waltham, MA, USA), and processed for analysis by mass spectrometry as described below.

### 4.6. Sample Preparation for Mass Spectrometry

For immunoprecipitation-derived samples, bead-bound proteins from immunoprecipitation experiments were digested and eluted by direct incubation with 50 ng trypsin in ABC overnight at 37 °C. The eluate was then reduced with dithiothreitol (DTT) and then alkylated with iodoacetamide (IAA). Formic acid and acetonitrile were added to final concentrations of 0.1% and 5% respectively. For gel slices, samples were destained in a buffer containing acetonitrile and ABC, reduced with DTT and then alkylated with IAA. The gel slices were dehydrated with acetonitrile, then resuspended with a trypsin digestion solution (100 ng trypsin per sample). Samples were digested overnight at 37 °C. All samples were cleaned with Aspire desalting tips (Thermo Fisher Scientific, New York, NY, USA) according to the manufacturer’s instructions as the final step before mass spectrometry analysis.

### 4.7. Mass Spectrometry

Mass spectrometry analysis was performed on an LTQ Orbitrap (Thermo Scientific) coupled to an Eksigent nano-LC Ultra HPLC (Absciex, Framingham, MA, USA). Samples were run on a 180 min (entire immunoprecipitation samples) or 30 min (gel slices) nonlinear gradient from 2% to 41% acetonitrile in 0.1% formic acid. Survey full-scan mass spectra were acquired from 300–2000 *m/z*, with a resolution of 60,000. The top 20 most intense ions from the survey scan were isolated and fragmented in the linear ion trap by collision-induced dissociation (normalized collision energy = 35 eV). The dynamic exclusion list (n = 500) used a retention time of 90 s and a repeat duration of 45 s (repeat count = 1), and preview scan mode was enabled. Ions of charge state = 1 or unassigned charge states were rejected.

### 4.8. Immunofluorescence Imaging

Cells were grown on fibronectin-coated coverslips (BD Biosciences), and fixed for 15 min in 4% paraformaldehyde followed by 10 min in cold methanol [90]. Primary antibodies were incubated for 1 h or overnight at the following concentrations: DDX6 (Santa Cruz Biotechnology, Dallas, TX, USA, sc-376433, 1:25), DCP1a (Abcam, ab47811), HA (Roche, 11867423001), NUFIP2 (Sigma, HPA017344, 1:250), GRAN1 (ProteinTech, 20808-1-AP, 1:250), GRAN2 (Sigma, HPA045542, 1:500), PABPC1 (Abcam, ab6125, 1:1000). Fluorescence-conjugated secondary antibodies were incubated for 1 h at a 1:1000 dilution (Molecular Probes, Waltham, MA, USA).

### 4.9. Bioinformatics and Data Analysis

Evidence for previously established protein-protein interactions was taken from the BioGRID and INTACT databases [55,103], and searches of the literature. Functional enrichment of gene lists by GO annotation was performed using the ProfCom_GO statistical framework [104]. Western blots were quantified via the ImageJ program using standard densitometry techniques [105].

### 4.10. Mass Spectrometry Data Analysis

RAW files from the mass spectrometer were converted to the mzXML format with ReadW (version 4.3.1, which is available in the TransProteomic Pipeline (TPP) platform (http://tools.proteomecenter-.org/software.php)) and then searched against a human proteome database (ENSEMBL 67) with X!Tandem/the Global Proteome Machine Cyclone XE (version 2.2.1 Beavis Informatics Ltd., Winnipeg, Canada) [106]. Searches were conducted using the following parameters: fragment monoisotopic mass error 0.4 Da, parent monoisotopic mass error plus −/+20 ppm, spectrum conditioning dynamic range 100 and total peaks 50, maximum parent charge 4, minimum parent M + H 500.0, minimum fragment *mz* 150.0, minimum peaks 15, cleavage site [RK] [107], maximum missed cleavage sites = 1 with a refinement step for unanticipated cleavage, complete carbamidomethylation of cysteines (+57 Da), partial oxidation of methionine (+16 Da), and partial deamidation of asparagine and glutamine (−1 Da) in the refinement step only. Protein abundance factor (PAF) was calculated as previously described [108], using an average of the three benzonase-treated replicates. The MS/MS data were also searched against a uniprot-based human protein sequence database including protein sequences of common contaminants by MaxQuant version 1.3.0.3. A FASTA file of the human reference proteome was obtained from UniProt (06-2012, 20,231 entries) [107]. For the searches, trypsin was defined as the protease. The search included carbamidomethyl of cysteine as a fixed modification and N-acetylation of protein and oxidation of methionine as variable modifications. Up to two missed cleavages were allowed for protease digestion and peptide had to be fully tryptic. The mass spectrometry proteomics data have been deposited to the ProteomeXchange Consortium via the PRIDE partner repository [109] with the data identifier PXD002070.

### 4.11. Sumoylation Bioinformatics Analysis

The software SUMOsp was used to identify sumoylation sites, with the “high-confidence” cutoff values [110]. Statistical significance of enrichment of both sumoylated proteins and sumoylation sites was calculated using hypergeometric distributions.

## 5. Conclusions

In sum, its multitude of interaction partners and memberships in protein complexes places DDX6 into a key position with a central role in RNA localization and metabolism. While several of the interaction partners have been known before, no study has placed DDX6 into the center of its interaction network, screening for interaction partners both in a comprehensive and highly specific way, minimizing the false-positive rates that are normally high. We present the first study, generate new hypotheses on the protein SUMOylation in DDX6 function and its putative role in splicing, and describe new stress granule components.

## Supplementary Files

Supplementary File 1
